# TGF-β_1_ As Possible Link between Loss of Bone Mineral Density and Chronic Inflammation

**DOI:** 10.1371/journal.pone.0014073

**Published:** 2010-11-22

**Authors:** Sabrina Ehnert, Johannes Baur, Andreas Schmitt, Markus Neumaier, Martin Lucke, Steven Dooley, Helen Vester, Britt Wildemann, Ulrich Stöckle, Andreas K. Nussler

**Affiliations:** 1 Department of Traumatology, MRI, Technische Universität München, München, Germany; 2 Department of Medicine II, University Hospital Mannheim, University of Heidelberg, Heidelberg, Germany; 3 Julius Wolff Institut, Berlin-Brandenburg Center for Regenerative Therapies, Charité-Universitätsmedizin Berlin, Berlin, Germany; The University of Akron, United States of America

## Abstract

**Background:**

The TGF family plays a key role in bone homeostasis. Systemic or topic application of proteins of this family apparently positively affects bone healing *in vivo*. However, patients with chronic inflammation, having increased TGF-β_1_ serum-levels, often show reduced bone mineral content and disturbed bone healing. Therefore, we wanted to identify intracellular mechanisms induced by chronic presence of TGF-β_1_ and their possible role in bone homeostasis in primary human osteoblasts.

**Methodology/Principal Findings:**

Osteoblasts were isolated from femur heads of patients undergoing total hip replacement. Adenoviral reporter assays showed that in primary human osteoblasts TGF-β_1_ mediates its signal via Smad2/3 and not Smad1/5/8. It induces proliferation as an intermediate response but decreases AP-activity and inorganic matrix production as a late response. In addition, expression levels of osteoblastic markers were strongly regulated (AP↓; Osteocalcin↓; Osteopontin↑; MGP↓; BMP 2↓; BSP2↓; OSF2↓; Osteoprotegerin↓; RANKL↑) towards an osteoclast recruiting phenotype. All effects were blocked by inhibition of Smad2/3 signaling with the Alk5-Inhibitor (SB431542). Interestingly, a rescue experiment showed that reduced AP-activities did not recover to base line levels, even 8 days after stopping the TGF-β_1_ application.

**Conclusions/Significance:**

In spite of the initial positive effects on cell proliferation, it is questionable if continuous Smad2/3 phosphorylation is beneficial for bone healing, because decreased AP-activity and BMP2 levels indicate a loss of function of the osteoblasts. Thus, inhibition of Smad2/3 phosphorylation might positively influence functional activity of osteoblasts in patients with chronically elevated TGF-β_1_ levels and thus, could lead to an improved bone healing *in vivo*.

## Introduction

Throughout life, bone undergoes continuous remodeling by a coordinated process of bone formation and bone resorption. Bone is formed by osteoblasts, which are of mesenchymal origin, and is resorbed by osteoclasts, derived from the hematopoietic system. Both actions are closely linked to maintain constant bone mass in the adult skeleton. Deregulation of this balance underlies the pathological loss of bone mass seen with delayed bone healing after fracture, osteoporosis and other metabolic bone diseases. Despite of their importance for our understanding of normal bone metabolism and the pathogenesis of metabolic bone diseases, the molecular mechanisms that govern the coordination of these processes are largely unknown.

Bone morphogenic proteins (BMPs), members of the transforming growth factor-β (TGF-β) superfamily, are able to promote osteogenesis, chondrogenesis and adipogenesis, whereas they inhibit myogenesis of mesenchymal progenitor cells [1]. However, as TGF-β is by far the most abundant cytokine in bone, by its mere abundance (200 µg/kg), it must be considered as a central player in bone turnover [2]. Both osteoblasts and osteoclasts secrete all three TGF-β iso-forms (TGF-β_1_, -β_2_ and -β_3_), which are present in their latent form within bone matrix [3], [4]. During bone turnover, acidification of the resorption lacuna by osteoclasts is thought to activate TGF-β [5], which should then stimulate the formation of bone [6]. Systemic or topic application of proteins of this family apparently positively affects bone healing *in vivo*. However, TGF-β is also strongly expressed during various inflammation reactions. Patients with liver fibrosis or cirrhosis often show elevated TGF-β levels over a long period [7], [8]. Similar results are seen in cardiac fibrosis, chronic renal failure or fibrosis of other tissues [9], [10], [11]. Thus, we propose that chronically increased serum levels of TGF-β_1_ observed in many systemic diseases might be a potential inducer for associated loss of bone density, as seen in hepatic or renal osteodystrophy.

Members of the TGF-β superfamily transduce their signals through two types of serine/threonine kinase receptors, termed type I and type II [12], [13]. The type II receptors are constitutively active kinases which phosphorylate type I receptors upon ligand binding. Seven type I receptors termed activin receptor-like kinase (Alk)-1 through -7 have been identified in mammals. BMPs, activins and TGF-β_1–3_ bind to different type I receptors, depending on the cell type. BMPs preferably bind to Alk2, -3 and -6, whereas activins and TGF-β_1–3_ bind to Alk-4 and Alk-5, respectively. Upon activation by type II receptors, Alks activate (phosphorylate) transcription factors, so-called Smads, in the cytoplasm. Eight different Smads have been identified in mammals. They are classified into three groups: receptor-regulated Smads (R-Smads/Smad1, -2, -3, -5 and -8), inhibitory Smads (I-Smads/Smad6 and-7) and the common-partner Smad (Co-Smad/Smad4). Alk1, -2, -3 and -6 activate Smad1/5/8 while Alk4, -5 and -7 activate Smad2/3 [14]. Upon activation Smad1/5/8 and Smad2/3 form complexes with Smad4, which allows them to translocate into the nucleus for regulation of target gene expression [15]. The signaling cascade of Smad2/3 can be inhibited by the chemical inhibitor SB431542, which has been shown to inhibit Alk4, -5, and -7 kinase activity specifically, but not Alk2, -3, and -6 kinase activity [16].

TGF-β_1_ is a multifunctional signaling protein that initiates a wide variety of responses in many different cell types. Thus, TGF-β_1_ is involved in embryogenesis, differentiation, wound healing, extracellular matrix (ECM) production and cell-cell adhesion [17], [18], [19]. Osteoblasts express a large variety of high affinity TGF-β receptors and therefore, TGF-β is thought to regulate many osteoblastic functions including expression of ECM genes, e.g. collagen and fibronectin, their integrin receptors and even the stabilization of integrin subunits [4], [20], [21], [22]. From the TGF-β superfamily, TGF-β_1_ showed the strongest chemotactic effect towards human osteoblasts, thus application of this cytokine in a dog model was able to enhance mechanical fixation, bone ingrowth and gap bone formation with unloaded implants surrounded by a gap. This effect was pronounced only with low concentrations of TGF-β_1_ but not with higher concentrations [23]. *In vitro* effects of TGF-β vary substantially depending upon the cell system. Neonatal and fetal organ cultures have generally indicated that TGF-β inhibits osteoclast differentiation from bone marrow monocytes, yet stimulates bone resorption by differentiated osteoclasts [24], [25]. From these results it is hard to draw conclusions for the normal role of TGF-β in bone development. Besides its complex and variable effects on bone cell populations *in vitro* and *in vivo*, a given experimental result with TGF-β may be potentially relevant to many different aspects of skeletal morphogenesis, including the generation of bone shape, bone growth, or bone remodeling. Thus, aim of this study was to investigate the long term effects of TGF-β_1_ on primary human osteoblasts in terms of signaling, proliferation, alkaline phosphatase (AP) activity, osteogenic marker gene expression and mineralized matrix formation.

## Results

### TGF-β_1_ increases proliferation but decreases AP activity in primary human osteoblasts

**Figure 1:**
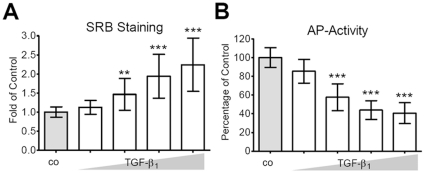
TGF-β_1_ increases proliferation but decreases AP activity in cultured osteoblasts. Primary human osteoblasts were treated for 8 days with different conc. (0, 1, 2.5, 5, 10 ng/ml) of hra TGF-β_1_. (A) SRB staining of surface proteins showed that TGF-β_1_ dose-dependently increased proliferation of osteoblasts. (B) In contrary, AP activity, normalized to relative cell numbers, was decreased by TGF-β_1_ in a dose-dependent manner. Results are expressed as mean ± standard deviation (N = 4, n = 4). **p<0.01, ***p<0.001 in comparison to untreated cells.

Primary human osteoblasts were treated with different conc. (0, 1, 2.5, 5, 10 ng/ml) of human recombinant active (hra) TGF-β_1_. After 8 days AP-activity was measured and adherent cells were fixed for SRB staining of surface proteins. SRB staining confirmed the microscopical observation that TGF-β_1_ induces proliferation in primary human osteoblasts in a dose dependent manner (Figure 1A). At the same time AP-activity was significantly reduced, dose-dependently (Figure 1B).

### TGF-β_1_ mediates its signal via Smad2/3 in primary human osteoblasts

**Figure 2:**
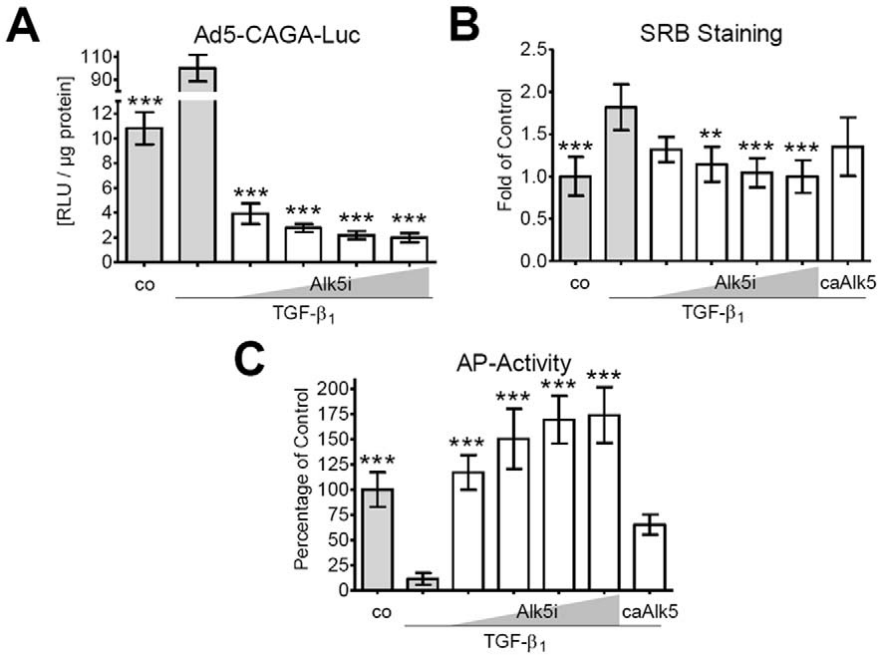
TGF-β_1_ mediated changes in primary human osteoblasts are Smad2/3-dependent. (A) Primary human osteoblasts (N = 4, n = 3) infected with Ad5-CAGA_9_-MLP-Luc reporter constructs (Smad2/4 reporter) were stimulated with 5 ng/ml hra TGF-β_1_ in the presence or absence of the Alk5 inhibitor SB431542 (5, 10, 20 and 40 nM). After 24 h luciferase activity was measured in cell lysates and normalized to total protein contents. (B/C) Primary human osteoblasts (N = 4, n = 4) were stimulated with 5 ng/ml hra TGF-β_1_ with or without the SB431542 (5, 10, 20, 40 nM) for 8 days or infected with Ad5-caAlk5 for 4 days. (B) Both TGF-β_1_ stimulation and caAlk5 infection increased proliferation (SRB staining) in primary human osteoblasts, which was inhibited dose-dependently by SB431542. (C) In the same line, AP activity, normalized to relative cell numbers, was decreased by TGF-β_1_ stimulation and infection with caAlk5. The decrease in AP-activity was blocked by SB431542 in a dose-dependent manner. Results are expressed as mean ± standard deviation. **p<0.01, ***p<0.001 in comparison to only TGF-β_1_ treated cells.

Primary human osteoblasts were infected with adenoviral reporter constructs (Ad5-CAGA_9_-MLP-Luc or Ad5-BRE-Luc) as described in materials and methods. After infection, cells were stimulated with 5 ng/ml hra TGF-β_1_. Cell lysates were taken after 24 h (Ad5-CAGA_9_-MLP-Luc) or 48 h (Ad5-BRE-Luc) and luciferase activity was measured. TGF-β_1_ increased only Smad3 regulated luciferase signal (Ad5-CAGA_9_-MLP-Luc) by 9.3-fold. Induction was completely inhibited by the Alk5 inhibitor SB431542, in a dose-dependent manner (Figure 2A). In contrast to BMP2 (1.97±0.18 fold; p<0.001) or BMP7 (2.24±0.21 fold; p<0.001), hra TGF-β_1_ was not able to induce Smad1 dependent luciferase expression.

### Increased proliferation and decreased AP-activity by TGF-β_1_ is dependent on Smad2/3 signaling and can be reversed by Alk5 inhibitor SB431542

Primary human osteoblasts were stimulated with 5 ng/ml hra TGF-β_1_ with or without different conc. of Alk5 inhibitor SB431542 (5, 10, 20, 40 nM) for 8 days. Cells were also infected with adenoviral constructs expressing constitutive active Alk5 (Ad5-caAlk5) for 4 days. In this setting, the constitutive active Alk5 ensures activation of the Smad2/3 pathway without additional stimulation with TGF-β_1_. AP-activity was measured and adherent cells were fixed for SRB staining of surface proteins. SRB showed that TGF-β_1_-dependent induction of proliferation in primary human osteoblasts can be inhibited by the Alk5 inhibitor SB431542 in a dose-dependent manner. Infection of cells with caAlk5 also led to increased SRB staining (Figure 2B). Interestingly, SB431542 not only inhibited the reduction of AP-activity, but even seemed to increase AP-activity dose-dependently (Figure 2C).

### Reversal of TGF-β_1_-dependent effects on AP-activity by SB431542 is time-dependent

**Figure 3:**
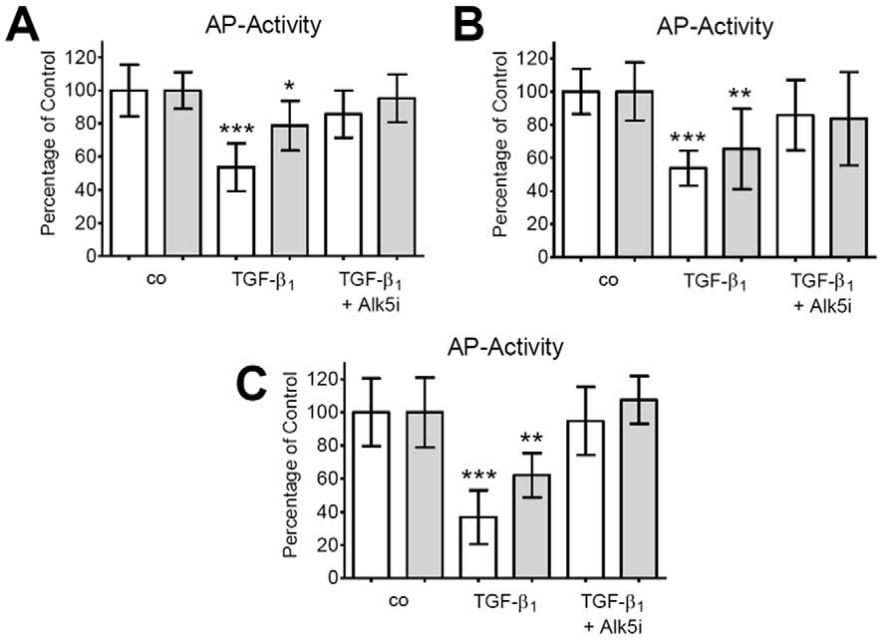
TGF-β_1_ dependent effects on AP activity by SB431542 are depending on time. Primary human osteoblasts stimulated with 5 ng/ml hra TGF-β_1_ in the presence or absence of 20 nM SB431542. After (A) 4, (B) 8 and (C) 12 days of continuous stimulation with TGF-β_1_ AP activity was measured and normalized to SRB staining for half of the cells (empty bars). The remaining cells were washed twice with DPBS and continued culturing for 8 days with basic culture medium. After the additional 8 days AP activity was measured in the same way (filled bars). Overall, with increasing time AP-activity was more and more reduced in the presence of TGF-β_1_. Supplementation with SB431542 was able to block the TGF-β_1_-dependent decrease of AP activity at all time-points. However, with increasing time of TGF-β_1_ pre-treatment, the so-called “rescue effect”, observed after the additional 8 days in basic culture medium, was reduced. Results are expressed as mean ± standard deviation (N = 4, n = 4). *p<0.05, **p<0.01, ***p<0.001 versus the corresponding untreated cells.

Primary human osteoblasts were stimulated with 5 ng/ml hra TGF-β_1_ with or without 20 nM Alk5 inhibitor SB431542. After 4, 8 and 12 days of continuous stimulation with TGF-β_1_ AP activity was measured for half of the cells. The other half of the cells was washed twice with DPBS and culturing was continued for 8 days with basic culture medium. After the additional 8 days AP activity was measured again. With increasing time AP-activity was further reduced by TGF-β_1_ treatment. For all time-points the Alk5 inhibitor SB431542 was able to block the TGF-β_1_-dependent decrease of AP activity. With increasing time of TGF-β_1_ pre-treatment, the so-called “rescue effect”, observed after the additional 8 days in basic culture medium, was reduced (Figure 3A–C).

### TGF-β_1_ inhibits formation of mineralized matrix in primary human osteoblasts

**Figure 4:**
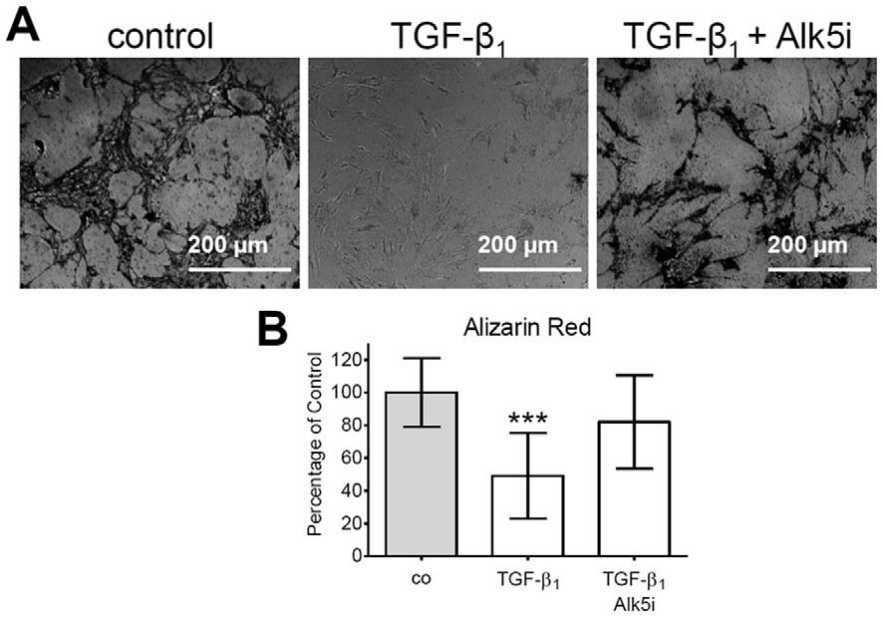
TGF-β_1_ inhibits formation of mineralized matrix in primary human osteoblasts. Primary human osteoblasts cultured in differentiation medium with 5 ng/ml hr TGF-β_1_ with or without 20 nM SB431542 for 20 days. (A) Representative picture for von Kossa staining for mineralized matrix. (B) Quantitative staining of produced mineralized matrix with Alizarin Red. Continuous stimulation of cells with TGF-β_1_ inhibited formation of mineralized matrix. We could prevent this effect by addition of SB431542. Results are expressed as mean ± standard deviation (N = 5, n = 6). ***p<0.001 in comparison to control conditions.

During osteogenic differentiation primary human osteoblasts were stimulated with 5 ng/ml hra TGF-β_1_ in the presence or absence of 20 nM Alk5 inhibitor SB431542. After 20 days mineralized ECM was stained with Alizarin Red or von Kossa. Von Kossa staining showed that constant treatment of primary human osteoblasts with TGF-β_1_ inhibited production of mineralized matrix (Figure 4A). Alizarin Red staining revealed that formation of mineralized matrix was reduced to 49.1±8.1% of control cells. This effect was partially reversed (81.9±8.0%) by the Alk5 inhibitor SB431542 (Figure 4B).

### TGF-β_1_ increases secretion of RANKL into the culture supernatant

RANKL levels in culture supernatants of human osteoblasts (N = 3, n = 4), stimulated for 8 days with 5 ng/ml hra TGF-β_1_ with or without 20 nM Alk5 inhibitor SB431542, were measured by ELISA. TGF-β_1_ increased RANKL secretion by 2.22±0.12 fold (p<0.001). Stimulation with SB431542 alone did not significantly alter RANKL levels in the culture supernatant (0.04±0.07 fold of control). However, SB431542 was able to significantly reduce the TGF-β_1_-dependant increase in RANKL secretion by 66.7±2.7% (p<0.001).

### TGF-β_1_ regulates expression of osteoblast marker genes

**Figure 5:**
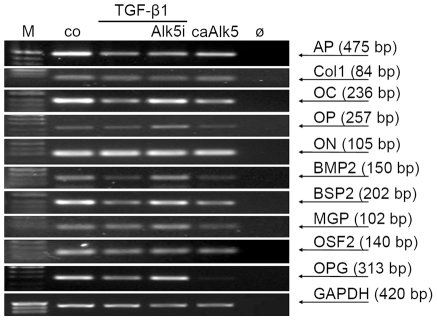
TGF-β_1_ regulates expression of osteoblast marker genes. Primary human osteoblasts (N = 4), stimulated for 8 days with 5 ng/ml hr TGF-β_1_ with or without 20 nM SB431542 or infected with Ad5-caAlk5 virus particles, were investigated for expression of osteogenic marker genes by RT-PCR. alkaline phosphatase (AP), collagen1 (Col1), osteocalcin (OC), osteopontin (OP), osteonectin (ON), BMP2, bone sialoprotein (BSP) 2, matrix gla protein (MGP), osteoblasts specific factor (OSF) 2 and osteoprotegerin (OPG). GAPDH was used as housekeeping gene. Ø negative control (H_2_O) Corresponding densitometric analysis is summarized in Table 2.

Primary human osteoblasts were stimulated with 5 ng/ml hra TGF-β_1_ with or without 20 nM Alk5 inhibitor SB431542. At the same time cells were infected with Ad5-caAlk5 virus particles. After 8 days we isolated mRNA for expression analysis. RT-PCRs were performed for AP, collagen1 (Col1), osteocalcin (OC), osteopontin (OP), osteonectin (ON), BMP2, bone sialoprotein (BSP) 2, matrix gla protein (MGP), osteoblasts specific factor (OSF) 2 and osteoprotegerin (OPG). GAPDH was used as housekeeping gene (Figure 5). Densitometric analysis (Table 1) showed that TGF-β_1_ treatment reduced AP mRNA levels compared to untreated cells, confirming results from AP activity measurement. In addition, expression levels of BMP2 and OSF2, involved in osteoblasts recruitment and adhesion, were reduced by TGF-β_1_ treatment and Ad5-caAlk5 infection. Col1 and ON mRNA levels were not significantly altered. Genes involved in matrix mineralization, e.g. OC, BSP and MGP, were significantly down-regulated by both treatments. In contrast, mRNA levels for OP, which favors osteoclast binding is increased by stimulation with TGF-β_1_ and over-expression of caAlk5. In the same line, mRNA levels of OPG, inhibiting osteoclast differentiation and activity, were reduced by both treatments. All observed effects were reversed by co-incubation with the Alk5 inhibitor SB431542.

**Table 1:** Densitometric analysis of RT-PCR.

Gene	Control	5 ng/ml TGF-β_1_	5 ng/ml TGF-β_1_ 20 nM SB431542	Ad5-caAlk5
AP	1.00±0.11	0.39±0.05	0.94±0.10	0.62±0.08
BMP2	1.00±0.16	0.57±0.21	1.11±0.13	0.37±0.04
OSF2	1.00±0.14	0.58±0.13	0.85±0.13	0.76±0.11
Col1	1.00±0.12	0.86±0.12	0.91±0.12	0.88±0.08
OC	1.00±0.16	0.40±0.06	1.02±0.17	0.51±0.11
ON	1.00±0.19	1.00±0.19	1.27±0.24	1.16±0.22
BSP2	1.00±0.14	0.22±0.03	1.61±0.25	0.47±0.09
MGP	1.00±0.10	0.83±0.10	1.38±0.19	0.65±0.07
OP	1.00±0.08	1.34±0.28	0.72±0.09	1.17±0.08
OPG	1.00±0.08	0.51±0.04	1.08±0.11	0.26±0.05

N = 4, n = 5.

### TGF-β_1_ reduces BMP2 and BMP7 mediated Smad1/5/8 signaling in human osteoblasts

**Figure 6:**
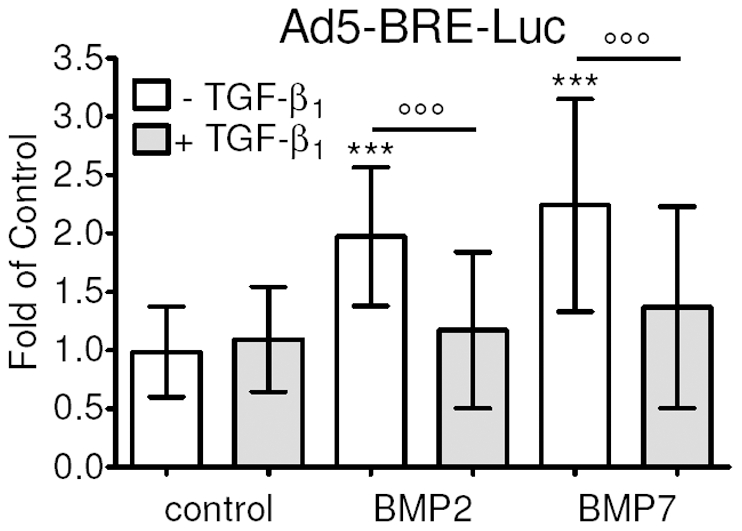
TGF-β_1_ suppresses BMP2 and BMP7 mediated Smad1/5/8 signaling in osteoblasts. Primary human osteoblasts infected with Ad5-BRE-Luc adenoviral particles (Smad1/4 reporter) were stimulated with 5 ng/ml hr TGF-β_1_ and/or 50 ng/ml hr BMP2 or -7. After 48 h luciferase activity was measured in cell lysates and normalized to total protein contents. BMP2 and -7 significantly induced Smad1/4 reporter signal by more than 2 fold. TGF-β_1_ showed now Smad1/4 reporter signal. Furthermore, in co-stimulation TGF-β_1_ even inhibited the observed BMP2 and -7 mediated Smad1/4 signaling. Results are expressed as mean ± standard deviation (N = 3, n = 6). ***p<0.001 versus untreated cells. °°°p<0.001.

Primary human osteoblasts were infected with Ad5-BRE-Luc adenoviral particles (Smad1/4 reporter construct) and stimulated with 5 ng/ml hra TGF-β_1_ and/or 50 ng/ml hra BMP2 or -7. Cell lysates were taken after 48 h and luciferase activity was measured. In contrast to BMP2 (2.0±0.2 fold) and BMP7 (2.2±0.2 fold), TGF-β_1_ failed to induce Smad1 regulated luciferase signal. Interestingly, BMP2 and -7 induced luciferase signal was blocked by co-incubation with TGF-β_1_ (Figure 6).

## Discussion

TGF-β is secreted by bone cells and therefore bone represents one of the biggest reservoirs for all three TGF-β iso-forms (TGF-β_1_, -β_2_ and -β_3_) of the human body. However, in bone matrix they are present in their latent form [4]. During bone resorption by osteoclasts, pH is decreased and this acidification is thought to activate the TGF-β reservoir which should then stimulate the formation of bone [5]. However, patients with chronic inflammation, often have constantly increased active TGF-β_1_ levels due to macrophage activation at the inflammation site. The distribution of this growth factor throughout the body via the bloodstream might influence other organs.

As osteoblasts contain a large variety of high affinity TGF-β_1_ receptors, TGF-β_1_ is thought to regulate many osteoblastic functions including expression of ECM genes, e.g. collagen and fibronectin, and the corresponding integrin receptors [4], [20], [21], [22]. Furthermore, it has been suggested that the down-regulation of cytokines and hormones, such as IGF-1, IL-11 and growth hormone, but also TGF-β1, is correlated with age related bone loss [26], [27], [28]. This hypothesis was supported by *in vitro* studies showing that TGF-β increases synthesis of type I collagen and non-collagenous bone proteins by cultured osteoblastic cells [2], [17], [29]. However, in our set-up of continuous TGF-β_1_ stimulation type I collagen mRNA did not alter. Moreover, matrix mineralization was blocked completely. In contrary, *in vivo* TGF-β_1_ knockout mice display a decrease of about 30% in tibia length and a reduction in bone mineral content [30], indicating the need for TGF-β_1_ in bone formation. Furthermore, local injection of TGF-β_1_ under the periosteum stimulated cartilage and bone formation [6], [31] while systemic injection of TGF-β_2_ leads to a general increase in osteoblastic activity [32]. In contrast, transgenic mice with osteoblasts specific (osteocalcin promoter) over-expression of TGF-β_2_ show a dramatic, age-dependent loss of bone mass similar to that seen in osteoporosis and hyperparathyroidism [33]. Furthermore, transgenic mice over-expressing osteoblast specific cytoplasmically truncated type II TGF-β receptor show decreased bone remodeling and increased trabecular bone mass with tougher femurs and stiffer and stronger vertebral bodies [34]. Similar results were observed when TGF-β type I receptor kinase was pharmacologically inhibited by SD-208. These mice showed increased bone mass and multiple aspects of bone quality as trabecular bone architecture and macro-mechanical behavior of vertebral bone [35]. Due to these, often contradictory, results we investigated the effects of TGF-β_1_ on primary human osteoblasts regarding proliferation, AP-activity, formation of mineralized matrix and osteogenic marker gene expression during a culture period of 20 days, thereby focusing on the underlying signaling cascades and mechanisms. Osteoblasts were isolated from femoral heads of patients undergoing total hip replacement. The average age of the patients was 75.25 years, which resembles the age of patients having an increased incidence of fractures because of loss of bone mineral density due to systemic disorders. In order to perform the experiments with a homogeneous population of primary osteoblasts we cultured and expanded our cells until passage 3 or 4 (up to 4 month) under standard culture conditions. Thus, the addition of TGF-β_1_ resembles the conditions observed in patients with chronic inflammation. During the first days TGF-β_1_ strongly induced proliferation of primary human osteoblasts. One possible mechanism might be an interaction between TGF-β_1_ signaling and Ras activation with subsequent stimulation of MAPKinases ERK, p38 and JNK [36]. This is supported by the *in vivo* findings showing that exogenously administered recombinant TGF-β was able to increase bone formation and to promote fracture healing [37]. TGF-β_1_, as well as PDGF-BB, showed the strongest chemotactic effects towards human osteoblasts and thus was able to enhance mechanical fixation, bone ingrowth and gap bone formation in a dog model with unloaded implants surrounded by a gap. Noteworthy, this effect was only present with low concentrations of TGF-β_1_ but did not appear any more with higher concentrations of this cytokine [23]. This is supported by our results showing that, although the observed increase in proliferation was more pronounced with increasing TGF-β_1_ concentrations, AP activity and formation of mineralized matrix was dose-dependently reduced. This effect was not only dose- but also time-dependent. Thus, a “rescue” experiment, documenting AP activity, showed that cells could not recover completely from TGF-β_1_ stimulation for more than 8 days. Thus, in patients with chronic inflammation, having significantly increased levels of circulating TGF-β_1_ for up to several years [7], [8], this might be a key regulator for reduced bone mineralization. Moreover, mRNA analysis revealed that gene expression of treated osteoblasts was shifted from osteoblasts maturation to osteoclast recruitment after only 8 days. For example osteopontin, favoring osteoclast binding, was induced by TGF-β_1_ in our cell system. Furthermore, we could show a significant increase in RANKL secretion when TGF-β_1_ is used as stimulus. At the same time OPG, an inhibitor for osteoclast differentiation and activity, was down-regulated in primary human osteoblasts. In contrast, in stromal cells TGF-β_1_ is reported to stimulate OPG and other factors that indirectly inhibit osteoclastogenesis [38]. Thus, it is conceivable that endogenous TGF-β is in the initial step essential for osteoclastogensis induced by a combination of receptor activator of NF-kB ligand (RANKL) and M-CSF [39]. Interestingly, TGF-β_1_ did not significantly alter Col1 expression in our experiments, but all genes involved in matrix mineralization, explaining the results observed by von Kossa and Alizarin Red staining for mineralized matrix. The observed effects of TGF-β_1_ could be inhibited by SB431542, which has been shown to inhibit ALK-4/5/7 kinase activity specifically, but not ALK2/3/6 kinase activity responsible for BMP signaling via Smad1/5/8 [16]. Therefore, we propose that the functional loss of osteoblast activity by TGF-β_1_ treatment is mediated via Smad2/3 signaling. This is supported by our findings that over-expression of constitutive active Alk5 induced similar effects as TGF-β_1_ in primary human osteoblasts. As expression of caAlk5 is independently of receptor binding activating Smad2/3 phosphorylation, our data clearly suggest that the observed effects are mediated by Smad2/3 signaling. Analysis of possible signaling cascades by adenoviral reporter assays revealed that TGF-β_1_ only activated Smad2/3 signaling but not Smad1/5/8 signaling as BMP2 or -7. Interestingly, BMP-mediated Smad1/5/8 signaling was reduced by TGF-β_1_ co-incubation, which might be an explanation for the observed functional loss of the osteoblasts. One possible mechanism for this inhibition might be increased expression of Smad6, as observed in TGF-β stimulated C2C12 cells [40].

As bone repair is one of the most important and urgent subjects for our over-aging society, members of the TGF-β superfamily, e.g. TGF-β_1_ and BMPs, are expected to be applied to the treatment of various orthopedic diseases including bone fracture and spinal fusion [18], [41]. Cell adhesion is the first crucial step for osseointegration as it specifies the fate of the cell. Furthermore, proliferation, differentiation, ECM production and organization as well as apoptosis are influenced by the events of cell-substrate adhesion [42], [43], [44]. However, as described before, effects of TGF-β vary substantially depending upon the cell system. While in neonatal and fetal organ culture TGF-β generally inhibited osteoclast differentiation from bone marrow monocytes, it stimulated bone resorption by differentiated osteoclasts [24], [25]. The normal role of TGF-β in bone development has been hard to infer from these studies. Besides its complex and variable effects on bone cell populations *in vitro* and *in vivo*, the exogenous application of TGF-β does not mimic its route of production within bone, where it is produced and stored largely as a bone matrix-bound latent complex that may be unable to induce cellular responses unless first released from mineralized bone during osteoclastic bone resorption [45]. TGF-β is strongly expressed during various inflammation reactions. Patients with liver fibrosis or cirrhosis often show elevated TGF-β levels [7], [8]. Similar results are seen in cardiac fibrosis, chronic renal failure or fibrosis of other tissues [9], [10], [11]. Thus, we propose that chronically increased serum levels of TGF-β_1_ observed in many systemic diseases might be a potential inducer for associated loss of bone density, as seen in hepatic or renal osteodystrophy. Thus, understanding the underlying mechanisms is mandatory to provide future possible therapeutic concepts for delayed fracture healing and metabolic bone diseases, e.g. osteoporosis, especially, as the reported *in vitro* and *in vivo* experiments administered TGF-β only for short times, which precludes an evaluation of the skeletal actions of TGF-β at steady state. Our study showed that despite the initial induction of proliferation, continuous stimulation of osteoblasts with TGF-β_1_ led to a functional loss which is Smad2/3-dependent. This knowledge opens new perspectives for investigation and treatment of delayed bone healing or metabolic bone diseases. For example local growth factor application to improve osteointegration can only be successful, if this initial recruiting and proliferation of osteoblasts does not interfere with BMP signaling as observed in the present experimental setup, representing constantly increased active TGF-β levels present in serum of patients with chronic inflammation, which is associated with functional loss of osteoblasts or osteoclast recruitment.

## Materials and Methods

Human recombinant active TGF-β_1_ (Peprotech, London, UK); Cell Culture Medium and supplements (PAA, Cölbe, Germany); Chemicals were obtained from Sigma (Munich, Germany).

### Ethics Statement

Osteoblasts were isolated from femur heads of patients undergoing total hip replacement, in accordance to the ethical vote of the MRI (“Ethikkommission der Fakultät für Medizin der Technischen Universität München”, http://www.ek.med.tum.de, Project Number 2413, TU Munich, Germany) and the patients' written consent. Bone tissue from (potential) tumor patients or patients with viral or bacterial infections was excluded from the study.

### Isolation and culture of primary human osteoblasts

We obtained femur heads from 8 patients (7 female, 1 male) with an average age of 75.25 years. Briefly, cancellous bone was removed mechanically from the femur head, washed 3–5 times with DPBS followed by 1 h incubation at 37°C with an equal volume of digestion buffer (DPBS, 0.07% Collagenase II – Biochrom AG, Berlin, Germany). After digestion, cancellous bone was washed with DPBS and transferred to cell culture flasks in culture medium (MEM/Ham's F12, 10% FCS, 2 mM L-glutamine, 100 U/ml penicillin, 100 µg/ml Streptomycin, 50 µM L-ascorbate-2-phosphate, 50 µM β-glycerol-phosphate). Medium was changed every 4–5 days. Within two weeks cells were growing out of the bone pieces [43], [46]. Osteoblasts were cultured and expanded until passage 3, where a pure population of osteoblasts was reached, as determined by flow cytometry, negative for CD14 and CD45 and positive for CD90 and CD105. Only cells in passage 3 and 4 were used for the experiments. For differentiation, cells were cultured for up to 20 days with differentiation medium (MEM/Ham's F12, 5% FCS, 2 mM L-glutamine, 100 U/ml penicillin, 100 µg/ml Streptomycin, 100 µM L-ascorbate-2-phosphate, 10 mM β-glycerol-phosphate, 25 mM HEPES, 1.5 mM CaCl_2_, 100 nM Dexamethasone). Medium was changed every 4^th^ day.

### Transient Cell Infections and Reporter Gene Assays

Cells were infected with the Smad1/4 reporter adenovirus (Ad5-BRE-Luc/provided from Prof. P. ten Dijke) or the Smad3/4 reporter adenovirus (Ad5-CAGA_9_-MLP-Luc) as described before [47]. Upon binding of phosphorylated Smad1/4 or Smad3/4, respectively, luciferase if expressed in the cytoplasm of the cells. Cell lysates and luciferase activity measurement was done according to the manufacturer's instructions, using the Steady-Glo Luciferase Assay System (Promega, Madison, USA) and normalized to total protein content.

Furthermore, we infected cells with adenoviral particles resulting in the expression of constitutive active Alk5 (Ad5-caAlk5), to investigate TGF-β effects independent of substrate binding. The expressed Alk5 is genetically modified in a way to constitutively activate Smad2/3 phosphorylation and associated signaling.

Infection efficiency was shown to be >90% by fluorescent microscopy of cells infected with Ad5-GFP (24 h).

### Alkaline Phosphatase (AP) Activity Measurement

Prior to substrate incubation with pNPP buffer (0.2% 4-nitrophenyl-phosphate disodium salt hexahydrate, 50 mM glycine, 1 mM MgCl_2_, 100 mM TRIS, pH 10.5) for 1 h, cells were washed with DPBS. Resulting formation of 4-nitrophenol (pNP), was determined photometrically at 405 nm. Signal was normalized to relative cell number determined by Sulforhodamine (SRB) staining as reported [48].

### Von Kossa and Alizarin Red Staining

Prior to fixation of cells with 100% ice cold ethanol (≥1 h), cells were washed with DPBS. Briefly, ethanol was removed by washing cells 3 times with tab water. For von Kossa staining cells were covered with staining solution (3% silver-nitrate) for 30 min at RT. To remove excessive staining solution, cells were washed 3 times with tab water. For color development (brownish-black) cells were covered with sodium-carbonate-formaldehyde solution (0.5 M sodium-carbonate, 10% formaldehyde). Pictures were taken with an inverted microscope. For Alizarin Red staining cells were covered with staining solution (0.5% alizarin red, pH = 4.0) for 30 min at RT. Excessive staining solution was removed by washing cells 3 times with tab water. Staining was resolved with 10% Cetylpyridiumchloride solution and resulting optical densities were measured at 562 nm [49]. Signals were normalized to relative cell number determined by Alamar Blue conversion (Biozol, Eching Germany).

### RANKL ELISA

RANKL levels in culture supernatants were measured by enzyme-linked immune-sorbent assay (ELISA) according to the manufacturer's protocol (Peprotech, London, UK).

### Conventional RT-PCR

Total cellular RNA was isolated with Trifast (Peqlab, Erlangen, Germany) according to the manufacturer's protocol. First-strand cDNA was synthesized from 1 µg total RNA using the Transcriptor High Fidelity cDNA synthesis kit (Roche, Mannheim, Germany). Primer information are summarized in Table 2. Products, resolved by gel electrophoresis in a 2% (w/v) agarose gel, were visualized with ethidiumbromide. Densitometric analysis of signals was performed using Image J software (NIH, Bethesda, USA).

**Table 2:** Summary of PCR conditions.

Gene	GeneBank accession[NM_]	Forward Primer 5′-3′	Reverse Primer 5′-3′	T_m_ [°C]	Product length [bp]
AP	000478.3	ACG TGG CTA AGA ATG TCA TC	CTG GTA GGC GAT GTC CTT A	53	476
Col1	000088.3	CAG CCG CTT CAC CTA CAG C	TTT TGT ATT CAA TCA CTG TCT TGC C	56	84
OC	199173.3	CCA.GCG.GTG.CAG.AGT.CCA.GC	GAC.ACC.CTA.GAC.CGG.GCC.GT	56	236
OP	000582	CTC CAT TGA CTC GAA CGA CTC	CGT CTG TAG CAT CAG GGT ACT G	60	257
ON	003118	AGC ACC CCA TTG ACG GGT A	GGT CAC AGG TCT CGA AAA AGC	60	105
BMP2	001200	CCC CCT ACA TGC TAG ACC TGT	CAC TCG TTT CTG GTA GTT CTT CC	60	150
BSP2	004967	TGA CTC ATC CGA AGA AAA TGG AG	CTG GAT TGC AGC TAA CCC TGT	60	202
MGP	000900	AGA TGG AGA GCT AAA GTC CAA GA	GTA GCG TTC GCA AAG TCT GTA	60	102
OSF2	006475	TAA GTT TGT TCG TGG TAG CAC C	GTG TGG GTC CTT CAG TTT TGA TA	60	140
OPG	002546.3	CCG GAA ACA GTG AAT CAA CTC	AGG TTA GCA TGT CCA ATG TG	60	313
GAPDH	002046.3	GTC AGT GGT GGA CCT GAC CT	AGG GGT CTA CAT GGC AAC TG	54	420

### Statistics

Results are expressed as mean ± standard deviation of at least 3 independent experiments (N≥3) measured as triplicates or more (n≥3). Data sets were compared by one-way analysis of variance (Kruskal-Wallis) followed by Dunn's multiple comparison test (GraphPad Prism Software, El Camino Real, USA). p<0.05 was taken as minimum level of significance.
